# The Association of Long-Functioning Hemodialysis Vascular Access with Prevalence of Left Ventricular Hypertrophy in Kidney Transplant Recipients

**DOI:** 10.1155/2014/603459

**Published:** 2014-01-28

**Authors:** Aureliusz Kolonko, Agata Kujawa-Szewieczek, Magdalena Szotowska, Piotr Kuczera, Jerzy Chudek, Andrzej Więcek

**Affiliations:** ^1^Department of Nephrology, Endocrinology and Metabolic Diseases, Medical University of Silesia, Francuska Street 20/24, 40-027 Katowice, Poland; ^2^Department of Pathophysiology, Medical University of Silesia, 40-752 Katowice, Poland

## Abstract

Left ventricular hypertrophy (LVH) is frequently observed in chronic dialysis patients and is also highly prevalent in kidney transplant recipients. This study evaluates the impact of long-functioning hemodialysis vascular access on LVH in single center cohort of kidney transplant recipients. 162 patients at 8.7 ± 1.8 years after kidney transplantation were enrolled. Echocardiography, carotid ultrasound, and assessment of pulse wave velocity were performed. LVH was defined based on left ventricular mass (LVM) indexed for body surface area (BSA) and height^2.7^. There were 67 patients with and 95 without patent vascular access. Both study groups were comparable with respect to gender, age, duration of dialysis therapy, and time after transplantation, kidney graft function, and cardiovascular comorbidities. Patients with patent vascular access were characterized by significantly elevated LVM and significantly greater percentage of LVH, based on LVMI/BSA (66.7 versus 48.4%, *P* = 0.02). OR for LVH in patients with patent vascular access was 2.39 (1.19–4.76), *P* = 0.01. Regression analyses confirmed an independent contribution of patent vascular access to higher LVM and increased prevalence of LVH. We concluded that long-lasting patent hemodialysis vascular access after kidney transplantation is associated with the increased prevalence of LVH in kidney transplant recipients.

## 1. Introduction

Left ventricular hypertrophy (LVH) is commonly observed in patients with chronic kidney disease [1] and constitutes an independent risk factor for cardiovascular events, the major cause of morbidity and mortality in end-stage renal disease (ESRD) patients [2, 3]. The etiology of LVH in ESRD is multifactorial and involves pressure overload (systemic, often poorly controlled hypertension and arteriosclerosis) and increased cardiac output (extracellular volume fluid expansion, arteriovenous vascular access (VA), and anemia) [4]. As a consequence, LVH is found in almost three-fourths of patients starting renal replacement therapy [5].

Following successful kidney transplantation, left ventricular mass (LVM) usually decreases, although the prevalence of LVH remains high [6, 7]. One of the factors that may contribute to the persistence of LVH after kidney transplant is the presence of functioning VA [8]. Such effects have been suggested by studies reporting the decrease of LVM or LVH prevalence after VA closure in hemodialysis [9] or kidney-transplanted patients [10–12] but negated by other authors [13, 14]. Also, the analyses of echocardiographic findings after kidney transplantation did not confirm the harmful role of VA [15, 16]. Thus, taking these inconsistencies into consideration, we have performed a study that aimed to evaluate the long-term impact of patent VA on LVH in ample cohort of kidney transplant recipients (KTRs).

## 2. Subjects and Methods

### 2.1. Study Participants

This study enrolled 162 KTRs, operated on in one center between 1998 and 2004, with known history of their VA and who were still attending our out-patient clinic between 2010 and 2011. The study protocol, adherent to Declaration of Helsinki, was accepted by local bioethics committee (KNW/0022/KB1/24/10), and all participants gave their written informed consent. In addition to data retrieved from the center-operated transplant patient registry, the study protocol comprised blood sample withdrawal during the routine ambulatory visit and the performance of echocardiography, carotid ultrasound, and the assessment of pulse wave velocity (PWV). All ultrasound studies were performed by one experienced sonographer.

Patients with a recent history of acute infection, cancer, or liver cirrhosis were excluded from this study. Additionally, to better separate the study groups, we excluded patients with their VAs closed surgically or spontaneously more than 2 years after transplant (*n* = 8).

Patients were scored as active smokers, when they were currently smoking or when they declared the period of nonsmoking as being shorter than 5 years.

Most patients received the immunosuppression therapy, based on cyclosporine (*n* = 110) or tacrolimus (*n* = 46), antimetabolic drug (mainly mycophenolate mofetil or mycophenolate acid), and steroids.

### 2.2. Clinical and Anthropometric Measurements

Weight and height were measured following standard procedures, and BMI was calculated in kg/m^2^. Body surface area (BSA), in m^2^, was calculated according to the DuBois formula (0.20247 × weight (kg)^0.425^  × height (m)^0.725^) [17].

Resting arterial blood pressure (BP) was measured three times in the sitting position on the arm without the vascular access, at the end of the physical examination. Patients with BP equal to or above 140/90 mmHg or those who received antihypertensive medication were diagnosed with hypertension.

Diabetes was determined in accordance with the American Diabetes Association criteria.

### 2.3. Laboratory Measurements

Routine laboratory measurements were performed in the hospital laboratory (Synchron Cx-9; Beckmann Coulter Inc., Fullerton, CA, USA). Serum high-sensitivity C-reactive protein (CRP) was measured using nephelometry (Siemens Healthcare Diagnostics, Deerfield, IL, USA), with a lower limit of sensitivity of 0.02 mg/L. Plasma concentrations of interleukin 6 (IL-6) and tumor necrosis factor-alpha (TNF-*α*) were measured using ELISA (R&D System, Minnesota, MN, USA). Serum N-terminal prohormone of the brain natriuretic peptide (NT-proBNP) was measured using electrochemiluminescence method (ECLIA), using commercially available kits (Roche Diagnostics GmbH, Mannheim, Germany) and Cobas E411 analyzer.

### 2.4. Echocardiography

Echocardiographic studies were performed using Acuson machine (Aspen, Mountain View, CA), equipped with 2.5–4.0 MHz micro-convex-array transducer. All subjects were examined using two-dimensional and M-mode echocardiography while lying down on their left side, using the parasternal long-axis sternal window. M-mode and 2D measurements were performed according to the American Society of Echocardiography recommendations [18]. These measurements included left ventricular end-diastolic (EDD) and systolic (ESD) diameters, intraventricular septum (IVS), and posterior wall (PW) end-diastolic thicknesses. Relative wall thickness (RWT) was calculated as follows: PW + IVS/EDD. A partition value of 0.42 for RWT was used for both male and female subjects.

### 2.5. Intima-Media Thickness

Carotid ultrasound was performed using Siemens machine (Sonoline Antares, Mountain View, CA), equipped with 4.0–9.0 MHz linear transducer. Carotid arteries were examined with a patient in the supine position with the neck extended. The evaluation included the common, internal, and external carotid arteries and the carotid bifurcation on each side. The common carotid artery intima-media thickness (IMT) was measured manually within 2 cm proximal to the carotid bulb, away from all identified plaques. The results from three separate measurements on each side were averaged.

### 2.6. Pulse Wave Velocity

The measurements were performed in the morning, after at least 15 min of rest in the supine position, using automatic noninvasive method (Complior, Colson AS, Paris, France). PWV was calculated as the time of pulse wave between the diagnosed points (distance (m)/time (s)). The signal of the pulse wave was received by a probe (TY-306, Fukuda, Tokyo, Japan) placed over the carotid and femoral arteries and connected with the automatic processor. All measurements were performed by one investigator.

### 2.7. Data and Statistical Analysis

LVM was calculated according to Devereux formula [19]. LVM was indexed separately for BSA (LVMI/BSA) and height (LVMI/height^2.7^). Thus, patients were diagnosed with LVH based on two separate criteria: LVMI/BSA > 110 g/m^2^ for women and >134 g/m^2^ for men [20] or LVMI/height^2.7^ > 45 g/m^2.7^ for women and >49 g/m^2.7^ for men [21].

Posttransplant major adverse cardiac events (MACEs) were defined as the episode of myocardial infarction, stroke, or cardiac artery stenting/surgery.

Plasma concentration of NT-proBNP was used as diagnostic marker of heart failure, as exemplified by the European Society of Cardiology Heart Failure Guidelines from 2008 [22]. A normal plasma NT-proBNP concentration (<400 pg/mL) has a high negative predictive value and makes diagnosis of heart failure unlikely, whereas concentration >2000 pg/mL makes the diagnosis very likely [22].

Kidney graft function was assessed by estimating glomerular filtration rate (eGFR), which was calculated according to abbreviated MDRD formula [23].

Statistical analyses were performed using STATISTICA 10.0 PL for Windows software package (StatSoft Polska, Kraków, Poland) and MedCalc 12.3.0.0. (Mariakerke, Belgium). Values are presented as means and 95% confidence intervals. For comparison of groups, we used the chi-squared test (qualitative variables) and ANOVA, followed by Tukey's test (quantitative variables). Correlation coefficients were calculated according to Pearson. Multivariate regression analysis (stepwise backward model) was performed for LVM, including potential explanatory variables: recipient age, gender, duration of pretransplant dialysis therapy, time after transplantation, eGFR, PWV, BMI, and the presence of hypertension and of patent VA. Factors potentially related to the occurrence of LVH were included into multiple logistic regression (stepwise forward model) analyses (i.e., recipient age, duration of dialysis therapy, time after transplant, eGFR, BMI, and the presence of hypertension and of patent VA). In all statistical tests, the “*P*” values below 0.05 were considered statistically significant.

## 3. Results

At the time of the study, there were 67 patients with functioning vascular access and 95 subjects without functioning VA. The majority of patients had VA located in the wrist region; only 9% and 5% of patients, respectively, had their VA located in cubital fossa. Of note, 21 patients without functioning fistula had never had this type of vascular access. Both study groups were comparable with respect to gender, age, duration of pretransplant dialysis therapy, and time after transplantation, kidney graft function, and cardiovascular comorbidities (Table 1).

**Figure 1:**
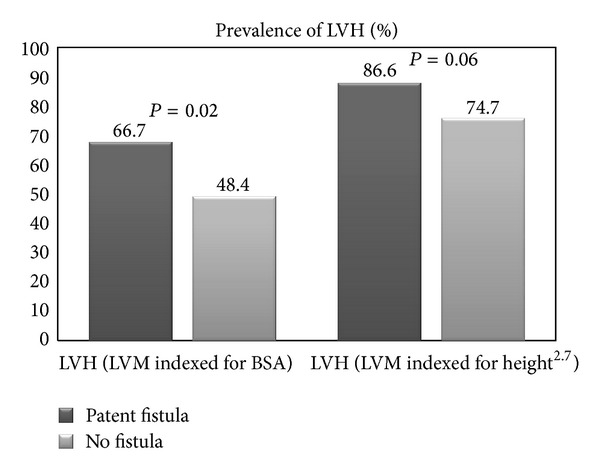


Patients with patent vascular access were characterized by significantly elevated LVM (Table 1) and greater percentage of LVH, based on LVM indexed for BSA (66.7 versus 48.4%, *P* = 0.02). The OR for LVH in patients with patent VA was 2.39 (1.19–4.76), *P* = 0.01. The occurrence of LVH based on LVM indexed for height^2.7^ was also higher in patients with patent VA (86.6 versus 74.7%, *P* = 0.06) (Figure 1) and with OR = 2.52 (0.99–6.47), *P* = 0.05. In the whole study population, the prevalence of LVH, based on LVM indexed for height^2.7^, was substantially larger than using LVM indexed for BSA (79.6 versus 55.9%, resp., *P* < 0.001). Pretransplant dialysis modality (hemodialysis versus peritoneal dialysis) had no significant influence on LVM and both LVM indexes in patients without patent VA.

Plasma concentrations of inflammatory markers (CRP and TNF-*α*) were similar in both groups, except for slightly higher plasma IL-6 concentration in patients with patent VA (4.82 (3.58–6.05) versus 3.42 (2.88–3.96) pg/mL, *P* = 0.02). There was no significant difference in plasma NT-proBNP levels between groups (432 (226–1012) versus 416 (210–1055) pg/mL (median values and quartiles)). The percentage of patients with plasma NT-proBNP concentration exceeding 2000 ng/mL was 12.1% in patent VA group and 6.8% in group without patent VA. However, the difference was statistically not significant.

Both LVM indices were significantly correlated with age (*r* = 0.281, *P* < 0.001, for LVMI/BSA, and *r* = 0.306, *P* < 0.001, for LVMI/height^2.7^), duration of pretransplant dialysis therapy (*r* = 0.192, *P* < 0.01, and *r* = 0.154, *P* < 0.05, resp.), time after transplantation (*r* = −0.232, *P* < 0.01, and *r* = −0.229, *P* < 0.05, resp.), NT-proBNP levels (for log values *r* = 0.436, *P* < 0.001, and *r* = 0.407, *P* < 0.001, resp.), eGFR (*r* = −0.155, *P* < 0.05, and *r* = −0.168, *P* < 0.05, resp.), and plasma TNF-*α* concentration (*r* = 0.183, *P* < 0.05, and *r* = 0.214, *P* < 0.05, resp.). Additionally, LVMI/BSA was associated with PWV (*r* = 0.163, *P* < 0.05), whereas LVMI/height^2.7^ was associated with CRP (for log values: *r* = 0.171, *P* < 0.05) and BMI (*r* = 0.405, *P* < 0.001). Of note, there was inverse correlation between eGFR and log NT-proBNP values (*r* = −0.513, *P* < 0.001).

In multivariate regression analysis, higher LVM was explained by age, male gender, longer duration of pretransplant dialysis therapy but shorter time after transplantation, larger BMI, worse kidney graft function, and the presence of patent VA (Table 2).

Logistic regression analysis also confirmed the independent contribution of patent vascular access to the presence of LVH calculated according to both indices used (Table 3).

## 4. Discussion

The regression of LVM after successful kidney transplantation is a well-established phenomenon [6–8]. However, it is not observed in all patients. In pediatric population, the efficient control of BP was shown to be an independent predictor for LVM index change after transplantation [7, 24]. The other important factors were pretransplant hemodialysis therapy, episodes of acute rejection, cumulative steroid dose, and anemia [25]. In the recently published prospective study on 37 adult renal transplant recipients without diabetes, the evolution of LVM after transplant was influenced by glucose metabolism disorders, markers of oxidative stress, and kidney graft function [26].

The results of our study have shown that the long-lasting presence of patent vascular access in KTRs is associated with the degree of LVH observed in this population. Moreover, the time after transplantation was inversely correlated with both LVMI/BSA and LVMI/height^2.7^, whereas longer posttransplant period independently predicted lower LVM, similar to higher eGFR. Of note, both groups did not differ in terms of demographic factors, duration of pretransplant dialysis therapy, and cardiovascular comorbidities. This may suggest that the beneficial effect of successful kidney transplantation on cardiac morphology may be fully expressed only in patients without patent VA. In contrary, patent vascular access had no effect on plasma NT-proBNP concentrations and only minor nonsignificant effect on the prevalence of heart failure diagnosed on the basis of plasma NT-proBNP levels.

Most previously published studies have analyzed the effect of VA closure on echocardiographic parameters in small subsets of KTRs, with relatively short followup [10, 11, 13–15]. Other reports have excluded patients with diabetes, low eGFR, ischemic heart disease, congestive heart failure, valvular heart disease, or proteinuria [12, 16, 27]. Taking into account the high prevalence of these comorbidities and their potential influence on LVH, the conflicting results of those studies remain inconclusive. Recently, Gorgulu et al. investigated a total of 130 renal transplant recipients, who were followed up for more than 6 months [16]. Despite some reports, which previously observed a significant decrease of LVM after VA closure within a similar period, one may speculate that such relatively short posttransplant period might, at least partially, explain their negative results [16].

In this study, we investigated the largest cohort of patients to date, and all patients were at least 6 years after transplant (mean 8.7 ± 1.8 years), with high prevalence of hypertension (85%). Of importance, we have examined all kidney graft recipients, transplanted between 1998 and 2004, who have still attended our transplant outpatient clinic in the time of the study. Moreover, two analyzed groups were clearly separated in terms of long-lasting fistula patency, by exclusion of those patients, in whom VA was functioning for more than 2 years after transplant and was surgically or spontaneously closed thereafter.

It is worth noting that there were no significant differences between groups in terms of PWV and IMT values and no differences in the incidence of coronary artery disease or cardiovascular adverse episodes observed before and after transplant, as well as in the prevalence and duration of arterial hypertension and the number of antihypertensive medications used at the time of examination. Thus, at the time of the study, there was no obvious evidence of worse status of the vascular system in patients with functioning VA. Furthermore, some patients from the group without patent vascular access were advised to close it surgically within the first 2 years after transplant, and they did so. Presumably, these patients had the highest blood flow and seriously increased cardiovascular risk; thus, they had the most to gain by closing the vascular access. Nevertheless, being several years after transplant, patients without patent vascular access had significantly lower LVM and lower prevalence of LVH, which could further suggest the particular importance of early VA closure for promoting the advantageous changes in cardiac morphology.

Limitations: unfortunately, in our study, we did not measure the vascular access blood flow in patients with patent VA or before VA closure; however, only 6.2% of all patients have had a fistula located within the cubital fossa, which is usually characterized by higher blood flow. The majority of patients had a patent or occluded vascular access within their wrist region. Additionally, we assume that the retrospective study design may be a source of bias, related to previous loss to followup of patients with severe LVH and heart failure, who died because of MACE during their first posttransplant years.

In conclusion, the long-lasting presence of patent vascular access after kidney transplantation is associated with the higher prevalence of LVH in these patients. As the multivariate regression analysis revealed the patency of vascular access as the only potentially modifiable independent variable, which may influence the prevalence of LVH in this population (the others were age, duration of pretransplant dialysis therapy, and eGFR), the closure of still functioning vascular access should be considered in patients with adequate kidney graft function, to reduce the risk of heart failure in long-term posttransplant period.

## Figures and Tables

**Table 1:** Characteristics of study patients with or without functioning vascular access (VA).

	Patent VA (*n* = 67)	No patent VA (*n* = 95)	Statistical significance
Demographic and anthropometric data			
Age (years)	49 (46–52)	50 (48–52)	0.58
Gender (M/F)	42/25	56/39	0.88
BMI (kg/m^2^)	27.2 (26.0–28.4)	26.7 (25.6–27.7)	0.51
BSA (m^2^)	1.93 (1.88–1.99)	1.88 (1.84–1.93)	0.15
Past medical history			
Duration of pretransplant dialysis therapy (years)	2.2 (1.9–2.8)	2.5 (2.1–2.9)	0.54
Time after transplantation (years)	8.0 (7.6–8.5)	8.7 (8.3–9.1)	0.08
Duration of hypertension therapy (years)	15.9 (14.3–17.5)	17.3 (15.6–18.9)	0.56
Hypertension (*n* (%))	55 (82.9)	83 (87.4)	0.35
Diabetes (*n* (%))	17 (25.4)	20 (21.1)	0.52
Symptomatic coronary artery disease (*n* (%))	12 (17.9)	16 (16.8)	0.97
MACE (*n* (%))	12 (17.9)	13 (13.7)	0.46
Active smokers (*n* (%))	12 (17.9)	20 (21.1)	0.62
Kidney graft function			
eGFR-MDRD (mL/min/1.73 m^2^)	50.8 (46.0–55.6)	51.4 (47.0–55.8)	0.82
Results of echocardiography			
ESD (mm)	30.2 (28.8–31.5)	29.3 (28.1–30.6)	0.35
EDD (mm)	50.6 (48.5–52.6)	48.5 (47.3–49.7)	0.008
IVS (mm)	12.5 (11.8–13.2)	11.9 (11.5–12.3)	0.09
PW (mm)	11.4 (11.0–11.8)	10.5 (10.1–12.8)	<0.001
LVM (g)	297 (269–324)	244 (228–260)	<0.001
RWT ≥ 0.42 (*n* (%))	43 (64.2)	60 (63.2)	0.97
LVM indexed for BSA (g/m^2^)	152 (139–165)	130 (121–138)	0.002
LVM indexed for height^2.7^ (g/m^2.7^)	69.6 (63.3–76.0)	59.3 (55.5–63.1)	0.003
Other measurements			
IMT (mm)	0.67 (0.63–0.72)	0.69 (0.65–0.72)	0.53
PWV (m/s)	13.0 (11.8–14.1)	12.4 (11.2–13.5)	0.48

Data shown as means ± 95% CI or frequencies. BMI: body mass index, BSA: body surface area, MACE: major adverse cardiac events, eGFR-MDRD: estimated glomerular filtration rate based on Modification of Diet in Renal Disease Study formula, ESD: left ventricular end-systolic diameter, EDD: left ventricular end-diastolic diameter, IVS: intraventricular septal thickness, PW: posterior wall thickness, LVM: left ventricular mass, RWT: relative wall thickness, IMT: intima-media thickness, and PWV: pulse wave velocity.

**Table 2:** The results of stepwise backward multivariate regression analysis for left ventricular mass (LVM).

Independent variable	*β*	*r* partial	*P*
Recipient age (per year)	1.91 ± 0.53	0.28	<0.001
Male gender	78.1 ± 12.3	0.46	<0.001
Duration of pretransplant dialysis therapy (per year)	6.57 ± 3.07	0.17	0.03
Time after transplantation (per year)	−0.61 ± 0.28	−0.17	0.03
eGFR (per 1 mL/min/1.73 m^2^)	−0.84 ± 0.29	−0.22	0.005
BMI (per 1 kg/m^2^)	5.67 ± 1.21	0.35	<0.001
Patent vascular access	43.6 ± 12.1	0.28	<0.001

Data shown as means ± 95% CI. LVM: left ventricular mass, eGFR: estimated glomerular filtration rate, and BMI: body mass index.

**Table 3:** The results of forward models of multivariate logistic regression analyses for left ventricular hypertrophy occurrence, measured based on both indices: LVMI/BSA and LVMI/height^2.7^.

Independent variable	LVH based on LVM indexed for BSA		LVH based on LVM indexed for height^2.7^	
	OR	*P*	OR	*P*
Age (per year)	1.04 (1.01–1.07)	0.009	1.05 (1.01–1.09)	0.02
Duration of pretransplant dialysis therapy (per year)	1.16 (0.98–1.39)	0.09	1.30 (1.00–1.69)	0.04
eGFR (per mL/min/1.73 m^2^)	0.98 (0.96–1.00)	0.03	—	
BMI (per 1 kg/m^2^)	—		1.22 (1.09–1.36)	<0.001
Patent vascular access	2.39 (1.19–4.76)	0.01	2.52 (0.99–6.47)	0.05

Data shown as means ± 95% CI. LVH: left ventricular hypertrophy, LVM: left ventricular mass, BSA: body surface area, eGFR: estimated glomerular filtration rate, and BMI: body mass index.
